# Vegetarian diet may ameliorate uremic pruritus in hemodialysis patients

**DOI:** 10.1080/0886022X.2018.1512871

**Published:** 2018-11-06

**Authors:** Chun-Yang Tseng, Tai-Te Wu, Chia-Wen Lai, Hsuan-Jen Lin, Che-Yi Chou, Chiz-Tzung Chang, Hung-Chih Chen

**Affiliations:** a Kidney Institute and Division of Nephrology, China Medical University Hospital, Taichung, Taiwan;; b Department of Health Care Management, Chang Gung University, Taoyuan, Taiwan;; c Division of Nephrology, Asia University Hospital, Taichung, Taiwan;; d Division of Nephrology, Asia University Hospital, Taichung, Taiwan;; e Division of Nephrology, Asia University Hospital, Taichung, Taiwan;; f Kidney Institute and Division of Nephrology, China Medical University Hospital, Taichung, Taiwan;; g Division of Nephrology, Asia University Hospital, Taichung, Taiwan

**Keywords:** Interleukin-2, hemodialysis, uremic pruritus, vegetarian diet

## Abstract

**Objectives:** Systemic inflammation has been reported to be associated with uremic pruritus (UP). Although a vegetarian diet can reduce systemic inflammation in hemodialysis patients, the effect of vegetarian diet on UP is not clear. The purpose of the study was to know the possible effects of vegetarian diet on UP.

**Methods:** A cross-sectional study was done to compare the severity of UP and blood levels of systemic inflammatory markers between vegetarian and non-vegetarian hemodialysis patients. Six non-vegetarian patients with uremic pruritus changed their non-vegetarian diet to vegetarian diet for 2 months. Visual Analogue Scale (VAS) and pruritus score (PS) were used to measure the UP severity. The serum high-sensitivity C-reactive protein (hs-CRP), and interleukin-2 (IL-2) were used as markers of inflammation.

**Results:** Both the median VAS scores (*p* = .043) and the median PS scores (*p* < .001) were lower in the Vegetarian than in the non-vegetarian group. The median values of hs-CRP in Vegetarian were lower than that for the non-vegetarian (*p* = .020). The median value of IL-2 was also lower in Vegetarian than that of the non-vegetarian (*p* = .016). There were 6 non-vegetarian patients shift to vegetarian for 2 months. The pruritus score improved and IL-2 level decreased after change to vegetarian diet.

**Conclusion:** We concluded that vegetarian diet might be associated with the amelioration of the uremic pruritus severity in hemodialysis patients.

## Introduction

Pruritus is a common and bothersome symptom among end-stage renal disease (ESRD) patients under hemodialysis (HD) treatment. While previous studies have shown that uremic pruritus (UP) can be present in up to 40% of hemodialysis patients [1], the pathophysiology of UP is incompletely understood. Dialysis clearance, metabolic factors, especially uremic hyperparathyroidism, iron deficiency anemia, neuropathy, medication, and skin xerosis have long been known as risk factors of UP [2–4]. Uremic pruritus has also been known to a systemic disorder associated with inflammation. High sensitivity C-reactive protein (hs-CRP) for example, is an inflammatory marker and has been associated with UP both in hemodialysis and peritoneal dialysis patients [5,6]. Recently, serum interleukin 2 (IL-2), has also been found to be elevated in hemodialysis patients with UP [7].

A vegetarian diet is known to be good for health. We have previously reported that hs-CRP was lower in uremic vegetarians on hemodialysis therapy than that of the uremic non-vegetarians on hemodialysis [8]. However, the effects of a vegetarian diet on UP are not known. We used serum hs-CRP and IL-2 as inflammatory and possible UP markers. We quantified the severity of pruritus with different scoring systems. The differences of these inflammatory markers and scores were compared between hemodialysis-treated vegetarians and non-vegetarians.

## Materials and methods

A cross-sectional study was performed. All the vegetarians or non-vegetarian uremia patients on a 4-h session thrice-weekly hemodialysis therapy for more than one year in two different dialysis centers were enrolled for our study. We excluded patients with abnormal liver function, hepatitis B or hepatitis C carrier, or patients under long-term steroid treatment secondary to systemic or dermal inflammatory disorders; these diseases perhaps will affect the severity of pruritus. A 24-h diet recall method was used for diet evaluation [8]. Vegetarians without consuming any meat, milk, or egg products were deemed strict vegetarian. Vegetarians taking also milk or egg products were deemed lacto-ovo vegetarian. All vegetarians started their vegetarian diet before the commencement of maintenance hemodialysis and all the vegetarians adhered to vegetarian diet more than one year. Both strict and lacto-ovo vegetarians were included into the vegetarian group.

Patients with serum potassium above 5.5 meq/L were given polystyrene sulfonic acid powder for potassium control. Calcitriol was used to control secondary hyperparathyroidism. All patients underwent hemodialysis treatment using high-flux artificial kidneys with low molecular weight heparin as anticoagulant. The quality of water for hemodialysis was monitored regularly following the standards of Association for the Advancement of Medical Instrument (AAMI) guidelines for dialysis water.

The blood sampling was performed before hemodialysis after getting inform consents of patients following regulations of the institutional review board. Patients’ fasting complete blood counts, serum biochemistry, electrolytes, and iPTH were checked. Serum hs-CRP was measured using the nephelometry methods. Serum IL-2 were examined using commercial ELISA kits according to the manufacturer`s manual (abcam^®^, Cambridge UK). All the samples were checked in triplicate. UP was evaluated by the subjective VAS (Visual Analogue Scale), and the pruritus score (PS) by different hemodialysis nurses as we previously described but with a minor modification [3–5]. In brief, the VAS measures the severity of pruritus with a reported score range from 0 to 10. Patients without UP have a score of 0 and patients with intolerable UP have a score of 10. PS grades the UP distribution, severity, frequency, and effects on sleep disturbances. For UP distribution, itch sensation confined to one spot receives one point, confined to one area 2 points, and generalized itch 3 points. For UP severity, itch sensation resolved without the need to scratch receives one point, itch sensation with the need to be scratched but with no degree of skin excoriation 2 points, itch scratched to the degree of skin excoriation 3 points, itch cannot be resolved even scratched to the degree of skin excoriation 4 points, and itch leading to restlessness 5 points. For UP frequency, itch sensation lasting shorter than 5 min and happening less than 4 times per day receives a score of one. Itch sensation lasting longer than 10 min each episode receives one point with a maximum of 5 points for this part. For the effects of UP on sleep, awakening due to pruritus gives 2 points per episode with a maximum of 6 points. The scores from above four parts (distribution, severity, frequency, effect on sleep disturbance) are summed up with a maximum score of 19. The antipruritic medication including oral anti-histamine and anti-pruritic ointments used by patients within previous one year were also recorded from medical chart review.

## Statistical analysis

IBM^®^SPSS^®^ Statistics version 21 for Macintosh was used for statistical analysis. Continuous variables were expressed as mean ± SEM (standard error mean), or median ± IQR (inter-quartile range). Student’s *t*-test was used generally used for comparison between groups, but for with marked skew distributions a Mann–Whitney test was applied. For categorical variables, Chi-Square or Fisher exact tests were used. Paired sample *t*-test used for compare biomarker and pruritus scale after diet change in cohort. A *p* value <.05 was deemed statistically significant.

## Results

### Demographic data

There were 241 patients receiving maintenance hemodialysis (4-h session hemodialysis therapy thrice-weekly) longer than one year, excluding those with hepatitis B, hepatitis C, and chronic steroid use. A total of 155 patents were enrolled for study.

Among these 155 patients, there were 15 vegetarian (Veg) and 140 non-vegetarian (non-Veg) hemodialysis patients. The two groups were age and sex matched, and there was no difference between hemodialysis vintage. There were 3 diabetes mellitus (DM) patients among the 15 Veg group and 48 DM among the 140 non-Veg group. The proportions of DM between these two groups were not statistically different (*p* = .388, by Fisher Exact test). Body mass index (BMI) of the non-vegetarian group was higher that of the in vegetarian group (*p* < .001), but the mean BMI of both groups can be maintained within the recommended reference range of 18.5–24.0 kg/m^2^ (Table 1).

**Table 1.** Demographic characters of non-vegetarian and vegetarian hemodialysis patients.

	non-Vegetarian (*n* = 140)	Vegetarian (*n* = 15)	*p*-value
Age (year)	58.5 ± 1.3	63.2 ± 2.5	.275
Male: Female	63:77	7:8	.510
HD vintage (months)	69.9 ± 5.2	71.2 ± 13.8	.924
DM: non-DM	48:92	3:12	.263
BMI (kg/m^2^)	22.4 ± 0.2	20.2 ± 0.5	<.001

HD: hemodialysis; DM: diabetes mellitus; BMI: body mass index.

### Blood test results

The hemoglobin and hematocrit were similar in both groups. The white blood cell count (WBC) was higher in non-Veg than Veg (6685 ± 211/μL vs 4870 ± 316/μL, *p* < .01). The serum ferritin of Non-Veg was similar to that of Veg. The normalized protein catabolic rate (nPCR), an indicator of protein intake, was lower in Veg patients (1.22 ± 0.28 g/kg/day vs 1.09 ± 0.31 g/kg/day; non-Veg vs Veg, *p* < .05). The serum albumin, AST, and ALT were not different between groups, as were as the levels of lipid profile. The BUN and serum creatinine were higher in non-Veg group (72.8 ± 1.8 mg/dL vs 61.2 ± 3.2 mg/dL; non-Veg vs Veg, *p* < .05 and 9.9 ± 0.2 mg/dL vs 8.6 ± 0.5 mg/dL; non-Veg vs Veg *p* < .05, respectively). The dialysis adequacy indicator Kt/V, however, was similar between these two groups. The serum uric acid was significantly higher in Non-Veg patients (7.1 ± 0.1 mg/dL vs 6.4 ± 0.3 mg/dL; non-Veg vs Veg, *p* < .05) Whereas, serum potassium and calcium were similar between these two groups, serum phosphate was lower in Veg (4.8 ± 0.1 mg/dL vs 4.2 ± 0.2 mg/dL; non-Veg vs Veg, *p* < .05), as was serum iPTH were in Veg patients (234.6 ± 33.3 pg/mL vs 113.8 ± 27.1 pg/mL; non-Veg vs Veg, *p* < .01). Alkaline phosphatase was similar between these two groups (Table 2).

**Table 2.** The blood counts and biochemical parameters of non-vegetarian and vegetarian hemodialysis patients.

	Non-Vegetarian (*n* = 140)	Vegetarian (*n* = 15)	*p*-value
Hemoglobin (g/dl)	10.6 ± 02	10.5 ± 0.4	.908
Hematocrit (%)	32.8 ± 0.4	32.4 ± 0.9	.784
WBC (/μL)	6685 ± 221	4870 ± 316	.004
Ferritin (ng/ml)	406.8 ± 32.6	498.4 ± 138.6	.614
Albumin (g/dl)	3.9 ± 0.3	3.8 ± 0.9	.186
AST (IU/L)	22.8 ± 0.8	26.4 ± 2.7	.164
ALT (IU/L)	19.7 ± 1.0	21.3 ± 3.0	.801
Triglyceride (mg/dl)	166.2 ± 9.8	164.3 ± 20.9	.936
Cholesterol (mg/dl)	168.3 ± 9.2	150.5 ± 18.5	.536
BUN (mg/dl)	72.8 ± 1.8	61.2 ± 3.2	.022
Creatinine (mg/dl)	9.9 ± 0.2	8.6 ± 0.5	.014
nPCR (g/kg/day)	1.22 ± 0.28	1.09 ± 0.31	.041
Kt/V	1.41 ± 0.28	1.48 ± 0.26	.865
Uric Acid (mg/dl)	7.1 ± 0.1	6.4 ± 0.3	.039
Potassium (mg/dl)	4.8 ± 0.1	5.0 ± 0.2	.345
Calcium (mg/dl)	9.9 ± 0.7	9.0 ± 0.3	.678
Phosphate (mg/dl)	4.8 ± 0.1	4.2 ± 0.2	.016
Ca x P	48.3 ± 3.8	38.9 ± 3.1	.141
iPTH (pg/ml)	234.6 ± 33.3	113.8 ± 27.1	.002
Alk-p (IU/L)	116.4 ± 6.4	101.6 ± 9.0	.458

WBC: white blood cells; AST: alanine aminotransferase; ALT: aspartate aminotransferase; BUN: blood urea nitrogen; nPCR: normalized protein catabolic rate; Ca x P: absolute value of calcium concentration multiplied by absolute value of phosphate concentration; iPTH: intact parathyroid hormone; Alk-p: alkaline phosphatase.

### Inflammatory markers and uremic pruritus severity

The median value of hs-CRP in Non-Veg was 0.75 (0.30, 1.26) mg/dL, which was significantly higher than that for the Veg 0.40 (0.30, 0.50) mg/dL (*p* = .020). Similar to the result for hs-CRP, the median value of IL-2 was also higher in Non-Veg 4.83 (2.1, 9.1) pg/mL than that of the Veg 2.53 (1.5, 3.2) pg/mL (*p* = .016).

The subjective pruritus scale VAS, which produces a score ranging from 0 to 10, was significantly higher in non-Veg [4.0 (1.6, 6.0)] than that of Veg [3.0 (0.0, 4.0)] (*p* = .022). The other pruritus scaling scoring system, PS, was also significantly higher in non-Veg [5 (4, 7)] than in Veg [4 (0, 5)] (*p* = .001) (Table 3).

**Table 3.** The inflammatory markers and pruritic scores of non-vegetarian and vegetarian patients receiving maintenance hemodialysis for more than one year.

	non-Vegetarian (*n* = 140)	Vegetarian (*n* = 15)	*p*-value
hs-CRP (mg/dL)	0.75 (0.30, 1.26)	0.40 (0.30, 0.50)	.020
IL-2 (pg/mL)	4.83 (2.08, 9.12)	2.53 (1.48, 3.19)	.016
VAS	4.0 (1.6, 6.0)	3.0 (0.0, 4.0)	.022
PS	5.0 (4.0, 7.0)	4.0 (0.0,5.0)	.001

hs-CRP: high sensitivity C-reactive protein; IL-2: interleukin 2; VAS: visual analog scale; PS: pruritus score.

### Anti-pruritic agents use

Anti-histamine oral pills or steroid-containing ointments are the most frequent medications used for pruritus. In parallel with the results of the above scoring, the frequency of anti-pruritic agents used within one year before blood testing was higher in non-Veg than in Veg. There were 59 patients in the 140 non-Veg had previously used oral anti-histamine in the past one year but there were only 2 patients who had previously used oral anti-pruritic agents in the 15 Veg (59/140 vs 2/15; non-Veg vs Veg, *p* = .020). Similar results can be seen in the numbers of patients who had used topical steroid ointment in the previous one year (55/140 vs 2/15; non-Veg vs Veg, *p* = .048). The median number of topical steroid ointments (clobetasol propionate 0.5 mg/tube) used by non-Veg was significantly higher than that of Veg [2 (0,4) tube vs 0 (0,0) tube; non-Veg vs Veg, *p* = .003, Mann–Whitney test] (Table 4). These data indicate that the severity of UP was greater in non-Veg than in Veg.

**Table 4.** The proportion of patients in non-vegetarian and vegetarian hemodialysis patients used oral, topical, or intravenous medication in the previous one year.

	non-Vegetarian (*n* = 140)	Vegetarian (*n* = 15)	*p*-value
Oral (patient number)^a^	59/140	2/15	.020
Topical (patient number)^a^	55/140	2/15	.048
Ointment (tube)^b^	0 (0,0)	2 (0, 4)	.003

a: Chi-square test; b: Mann–Whitney test.

### Non-diabetic hemodialysis patients

As diabetic hemodialysis patients are frequently have uremic pruritus, we then excluded diabetic hemodialysis patients and did further analysis. There were 92 non-diabetic non-vegetarian and 12 non-diabetic vegetarian hemodialysis patients. The hs-CRP was lower in non-diabetic vegetarians ([0.40 (0.30, 0.50) mg/dL vs 0.75 (0.30, 1.19) mg/dL, *p* = .049]). The IL-2 level of non-diabetic non-vegetarian [4.89 (2.21, 8.95) pg/mL] was higher than that of non-diabetic vegetarian [2.35 (1.17, 3.05) pg/mL] (*p* = .014). The VAS of non-diabetic non-vegetarian was higher than VAS of non-diabetic vegetarian [4.0 (1.6, 7.0) vs 2.0 (0.0, 3.7) non-Veg vs Veg, *p* = .030], and the PS of non-diabetic non-vegetarian was also higher than PS of non-diabetic vegetarian [5.0 (4.0, 8.0) vs 3.0 (0.0, 4.8) *p* = .003] (Table 5).

**Table 5.** The serum inflammatory markers and pruritic scores of non-diabetic non-vegetarian and non-diabetic vegetarian receiving maintenance hemodialysis for more than one year.

	non-Vegetarian (*n* = 92)	Vegetarian (*n* = 12)	*p*-value
hs-CRP (mg/dL)	0.75 (0.30, 1.19)	0.40 (0.30, 0.50)	.049
IL-2 (pg/mL)	4.89 (2.21, 8.95)	2.35 (1.17, 3.05)	.014
VAS	4.0 (1.6, 7.0)	2.0 (0.0, 3.7)	.030
PS	5.0 (4.0, 8.0)	3.0 (0.0, 4.8)	.003

hs-CRP: high sensitivity C-reactive protein; IL-2: Interleukin 2; VAS: visual analog scale (0–10); PS: pruritus score (0–19).

### Diet change

To further demonstrate the effect of vegetarian diet on inflammatory markers and its possible effects on UP, six hemodialysis patients with uremic pruritus shifted their non-vegetarian diets to vegetarian diets for 2 months. Their blood hs-CRP and IL2 levels decreased after diet changed and so did the UP severity scores (Table 6).

**Table 6.** The values of inflammatory indicators and pruritus severity in 6 non-vegetarian hemodialysis patients and after their changing to vegetarian diets for 2 months.

	Vegetarian diet	non-Vegetarian diet	*p*-value
hs-CRP (mg/dL)	1.90 ± 0.29	0.64 ± 0.09	<.001
IL-2 (pg/ml)	8.0 ± 0.6	2.9 ± 0.2	.003
VAS	8.8 ± 0.5	3.0 ± 0.2	<.001
PS	8.8 ± 0.4	3.8 ± 0.3	<.001

hs-CRP: high sensitivity C-reactive protein; IL-2: interleukin 2; VAS: visual analog scale (0–10); PS: pruritus score (0–19).

## Discussion

The current study findings demonstrate that the hemodialysis patients on a vegetarian diet were less likely to have uremic pruritus. In HD patients, the severity of uremic pruritus for the vegetarians, determined by both subjective and objective scoring methods, was lower than that of the non-vegetarians. The percentage of patients using anti-pruritic agents was also lower than that of the non-vegetarians. Finally, vegetarian hemodialysis patients had lower levels of hs-CRP and IL-2 than those of the non-vegetarian hemodialysis patients. When exclude diabetes patients for subgroup analysis, the above findings can also be applied to non-DM hemodialysis patients. The above study results were further validated by a small-scale cohort study.

The cause of UP is still not clear. Uremic toxin, inadequate dialysis, hyperphosphatemia, secondary hyperparathyroidism, iron deficiency anemia, heparin, biocompatible membrane, and skin xerosis have all been postulated as possible mechanism [9]. In our study, mean Kt/V, heparin, and biocompatible membrane use were similar in both groups. Thus, dialysis clearance, heparin, and artificial kidney may not be a key factor of UP difference between Veg and non-Veg group. Serum phosphate and iPTH were lower in our vegetarian patients, but the mean serum phosphate level and iPTH of the non-vegetarian patients were within an acceptable range [10]. The reason for higher serum phosphate in non-vegetarian patients was not clear. As non-vegetarians are more liberal for daily diet selection, they are more inclined to eat processed and convenience foot rich in phosphate contents. The higher serum phosphate led to higher calcium-base phosphate binder use and resulted in higher serum calcium and iPTH in non-vegetarians. There were several studies reported that serum phosphate levels and iPTH did not associate with pruritus [11,12]. It seemed that the differences between serum phosphate and iPTH were not able to fully explain the UP differences between these two groups.

Previous studies have suggested an association of UP with systemic inflammatory process as manifested by high serum hs-CRP in UP patients [13]. Recently serum IL-2, a systemic inflammatory cytokine, has also found to be elevated in UP patients [7]. IL-2 is a pruritic cytokine secreted by T helper 1 (Th1) cells and systemic IL-2 injection can result in pruritus [14,15]. The number of blood Th1 cells is higher in hemodialysis patient with UP than that of hemodialysis patients without UP. Deranged Th1 cells differentiation has also been postulated to be one of the possible mechanisms [16]. UVB exposure, which has a pronounced effect on Th1 and Th2 lymphocyte differentiation to attenuate Th1 expression, can relief of UP in a considerable number of patients [17]. Thalidomide, which can suppress TNF-α production and leads to a predominant differentiation of Th2 lymphocytes with suppression of IL2 producing Th1 cells, is effective in the therapy of UP [18]. These two UP treatment methods demonstrated the possibility of Th1 and IL-2 in the pathogenesis of UP. We did not check the absolute Th1/Th2 counts in our study. Although serum IL-2 has been found to be elevated in hemodialysis with UP, there was no significant linear correlation between IL-2 and UP severity [7]. This is compatible with our results (data not shown).

White blood cell count, another systemic inflammatory marker, was significantly lower in vegetarian patients. This result was similar to that in patients of rheumatoid arthritis, whose white blood cell count decreased after one-year of vegetarian diet [19]. Ferritin, which is an acute phase protein, cannot be a good indicator of systemic inflammation because of the frequent iron supply in hemodialysis patients. Furthermore, serum ferritin level of these two groups was within range suggested by DOQI guideline [20]. Higher CRP, higher IL-2, and higher white blood cell count all implicate the importance of system inflammation and UP.

Enhanced systemic inflammation in dialysis patient may be related to process associated with renal failure itself, may be a consequence of the treatment for renal failure (dialysis-related), or maybe even unrelated to either renal failure or the dialysis process specifically. There were studies showed that renal failure can cause accumulation of pro-inflammatory compounds and the products of metabolism in renal failure can reduce plasma antioxidant activity [21,22].

Long-term vegetarians have lower oxidative stress and systemic inflammation [23]. Vegetarian diet has been reported to alleviate T-cell mediated inflammatory skin disease [24]. We have previously also reported that blood systemic inflammation markers such as hs-CRP and white blood cell count were lower in vegetarian hemodialysis patients than in non-vegetarian hemodialysis patients [8]. It is reasonable to infer that hemodialysis patients on a vegetarian diet may also have a lower T-cell inflammation as well as its down-steam IL-2 secretion. This may help in explaining the improvement of UP that we saw in our study group. Recently, vegetarian diet in hemodialysis patients has been found to be able decrease the production of indoxyl sulfate, another possible marker of uremic pruritus [25]. We did not check the indoxyl sulfate level of our study subjects and the correlation of IL-2 with indoxyl sulfate was not known either.

In our study, it is limited by small patient number and difficulty for patients to shift from non-vegetarian to vegetarian diets. The study results can be biased by the small case numbers and relatively small size number in vegetarian diet changing (from non-vegetarian to vegetarian). The possible benefits of vegetarian diet versus non-vegetarian diet needs to be further evaluated in more randomized double-blind placebo-controlled trials with larger sample sizes.

## Conclusion

Hemodialysis patients on a vegetarian diet have a lower severity of uremic pruritus. The biomarkers of uremic pruritus (serum hs-CRP and IL-2) and severity of pruritus (scale by VAS and PS) are lower in vegetarian diet patients and vegetarian diet conversion. Vegetarian diet might be associated with the amelioration of the uremic pruritus severity in hemodialysis patients.
